# Cuttlefish *Sepia officinalis* Preferentially Respond to Bottom Rather than Side Stimuli When Not Allowed Adjacent to Tank Walls

**DOI:** 10.1371/journal.pone.0138690

**Published:** 2015-10-14

**Authors:** Darcy A. A. Taniguchi, Yakir Gagnon, Benjamin R. Wheeler, Sönke Johnsen, Jules S. Jaffe

**Affiliations:** 1 Scripps Institution of Oceanography, University of California, San Diego, La Jolla, California, 92093, United States of America; 2 Biology Department, Duke University, Durham, North Carolina, 27708, United States of America; University of Sussex, UNITED KINGDOM

## Abstract

Cuttlefish are cephalopods capable of rapid camouflage responses to visual stimuli. However, it is not always clear to what these animals are responding. Previous studies have found cuttlefish to be more responsive to lateral stimuli rather than substrate. However, in previous works, the cuttlefish were allowed to settle next to the lateral stimuli. In this study, we examine whether juvenile cuttlefish (*Sepia officinalis*) respond more strongly to visual stimuli seen on the sides versus the bottom of an experimental aquarium, specifically when the animals are not allowed to be adjacent to the tank walls. We used the Sub Sea Holodeck, a novel aquarium that employs plasma display screens to create a variety of artificial visual environments without disturbing the animals. Once the cuttlefish were acclimated, we compared the variability of camouflage patterns that were elicited from displaying various stimuli on the bottom versus the sides of the Holodeck. To characterize the camouflage patterns, we classified them in terms of uniform, disruptive, and mottled patterning. The elicited camouflage patterns from different bottom stimuli were more variable than those elicited by different side stimuli, suggesting that *S*. *officinalis* responds more strongly to the patterns displayed on the bottom than the sides of the tank. We argue that the cuttlefish pay more attention to the bottom of the Holodeck because it is closer and thus more relevant for camouflage.

## Introduction

Cuttlefish are capable of some of the most dynamic camouflage responses in the animal kingdom. They can change their body patterns quickly because the chromatophores in their skin are under direct neural control [1,2]. In addition to coloration, cuttlefish body patterning includes movement, posture, and texture, along with the use of reflective elements such as iridophores and leucophores [1]. They use this varied and complex body pattern repertoire to avoid detection by predators by resembling the diverse natural environments in which they live and also to avoid recognition by mimicking other objects or disrupting their body outline.

Despite the diversity of their camouflage response, most forms of cuttlefish body patterning have been previously classified into three general categories: uniform, mottled, and disruptive [1]. Uniform or uniformly stippled patterning consists of little to no contrast with light coloration displayed evenly across the mantle. Cuttlefish are uniformly colored when exposed to substrates such as sand, low-contrast patterns, or solid-colored backgrounds [3,4]. Mottled is characterized by larger, higher contrast elements than stippled, and the overall body tone can be either light or dark. This camouflage pattern is seen when the animals are exposed to small pebbles or a high density of contrasting elements such as checkerboard patterns [5–8]. Disruptive body patterning consists of relatively large components that vary in color, contrast, and orientation and serve to break up the outline of the animal. This body patterning can be seen when cuttlefish are exposed to such surfaces as rocks, shells, and large, high-contrast patterns [3,4,6,8,9]. These diverse body patterning responses are driven by visual stimuli; they can change their appearance without any tactile information [1,10–12]. Several elements within their environment are important for informing each body pattern response [13]. For example, cuttlefish are sensitive to the size of repeating elements within patterns [9], specifically relative to their own body size [6]. Cuttlefish also use the contrast and edges of visual patterns to help dictate their camouflage response [9,10,14,15]. Other visual information important for influencing body patterning include, but are not limited to, the area of patterns [16], number of contrasting elements [3,7,9], spatial frequency [17], and visual depth [12].

Because cuttlefish have a wide field of view, it is not immediately clear from where in their environment they are gathering these visual cues. However, several studies have shown that cuttlefish change their body pattern in response to visual stimuli that are displayed on tank walls, 3-dimensional objects, and substrata. For example, cuttlefish responded to the visual information described above when it was displayed on the substrata. The animals also lift their arms to mimic the angle of square wave stimuli presented on their tank walls, that is, side stimuli [11].

Researchers have also performed experiments to see if cuttlefish respond preferentially to patterns presented *either* on the sides or bottom of their tank, rather than just one in isolation. For example, Hanlon and Messenger [1], using uniformly colored stimuli, found that cuttlefish respond to the bottom of the tank rather than the sides. Barbosa et al. [18], using both a uniform color and checkerboard pattern, found that the latter on either the bottom or sides of the tank elicited disruptive coloration. Also, there was a stronger response when the patterns were presented on both the bottom and sides. Buresch et al. [19] specifically tested whether cuttlefish prefer to match their substrate or a 3D object placed in the experimental aquarium. Cuttlefish chose to masquerade as 3D objects but only when those objects were high-contrast and the substrate was low-contrast. To address more fully cuttlefish response to the bottom versus the sides of a tank, Ulmer et al. [20] investigated the extent of disruptive coloration in cuttlefish exposed to checkerboard patterns on 3D objects and the arena walls. Overall, they found that checkerboard patterns on 3D objects or walls were more influential than stimuli on the bottom in eliciting a disruptive patterning.

Synthesizing these studies, cuttlefish seem to respond predominantly to the sides and 3D objects rather than the substrate. However, in all of the above studies, the animals were allowed to move to the walls and 3D objects. Therefore, there remains the potentially confounding issue of whether the animals are preferentially responding to the walls and 3D objects or if instead the animals are simply responding to these vertical stimuli when they are close to them. That is, it is not clear if cuttlefish still respond to walls when they are distanced from them. Furthermore, in most of the above studies, only the extent of uniform and disruptive coloration was examined; cuttlefish were only exposed to nearly uniform colors and patterns and checkerboard patterns of one size.

**Fig 1:**
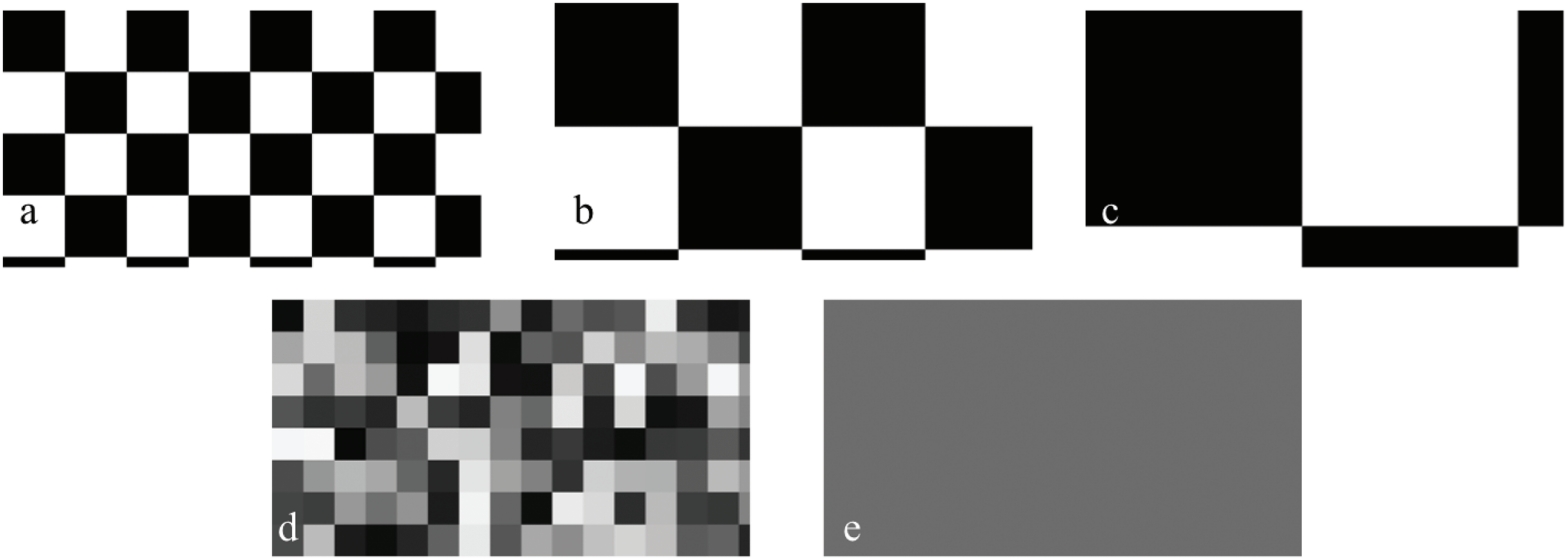
Stimulus patterns used for the tank bottom and side stimuli. (a) Small checkerboard, (b) medium checkerboard, (c) large checkerboard, (d) television static, and (e) uniform grey. In all images, only a portion of the pattern is shown rather than the entire tank wall or tank bottom.

**Fig 2:**
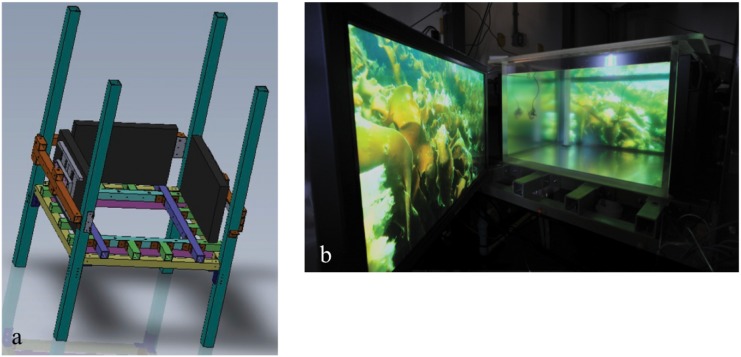
Sub Sea Holodeck. a. Schematic rendering of the Holodeck, with only three sides shown for clarity. b. Actual image of the Holodeck, showing the vertical planes adjacent to the tank sides. The horizontal stimulus DLP screens are not shown. Both images taken from [21]. Reprinted from [21] with permission from IEEE, original copyright 2015.

Here, we extend this previous research by examining the extent of uniform, disruptive, and mottled camouflage responses of cuttlefish exposed to various stimuli and by determining whether cuttlefish respond more to stimuli on the bottom or sides of an experimental tank when they are not allowed to settle next to the tank walls. The bottom stimulus refers to a pattern on the bottom of the tank, and the side stimulus refers to patterns projected simultaneously on all the sides of the tank. In this study, we exposed the cuttlefish *Sepia officinalis* (Linnaeus) to five distinct stimulus patterns: small, medium, and large checkerboards, television static, and uniform patterning (Fig 1). We restricted the cuttlefish to the middle of a novel aquarium setup, the Sub Sea Holodeck (Fig 2 [21]), and examined how variable the cuttlefish coloration was while the bottom and side stimuli were changed. We were thus able to test the hypothesis that cuttlefish still respond to the tank sides when not adjacent to them.

**Fig 3:**
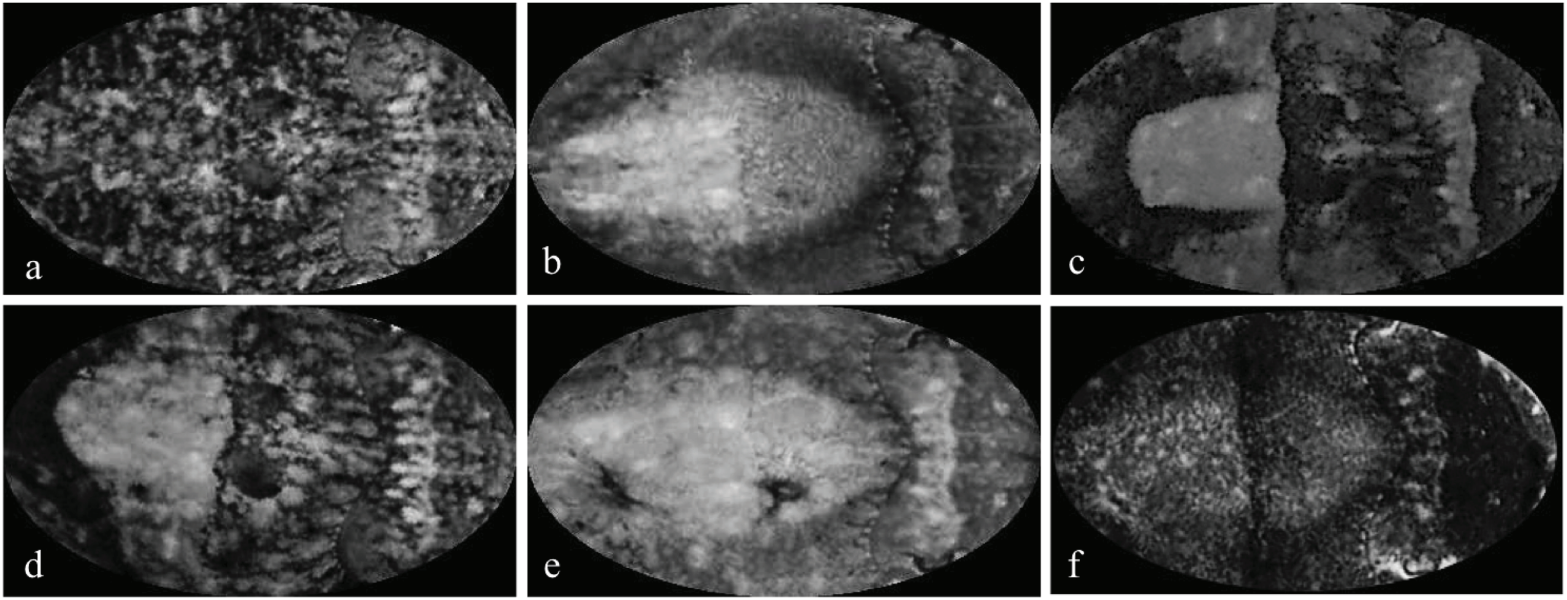
Example images used for camouflage categorization of segmented cuttlefish images. (a) 100% mottled, (b) 100% uniform, (c) 100% disruptive, (d) 50% mottled and 50% disruptive, (e) 50% mottled and 50% uniform, (f) 50% uniform and 50% disruptive.

To analyze the cuttlefish responses, we manually classified them into varying degrees of uniform, mottled, and disruptive. Cuttlefish responses were classified into combinations of two of the three patterns of uniform, mottled, and disruptive. The extent of categorization into each of these groups was chosen in increments of 0%, 25%, 50%, 75%, or 100%, summing to 100% (Fig 3). For example, a response could be classified as 25% mottled and 75% uniform or 50% uniform and 50% disruptive. This classification scheme resulted in twelve possible categories. We acknowledge that this method of classification limits the full range of potential camouflage patterning responses. However, as also mentioned in the *Materials and Methods* section, we aimed to provide enough flexibility to adequately distinguish the cuttlefish responses while also avoiding making the classification unnecessarily complex, the latter of which could lead to inaccurate classifications. In addition, given our objective of comparing cuttlefish camouflage responses to the bottom *versus* the side stimuli, the classification technique, as long as it is consistent across all images, is somewhat arbitrary. Therefore, we also checked for consistency in classification.

**Fig 4:**
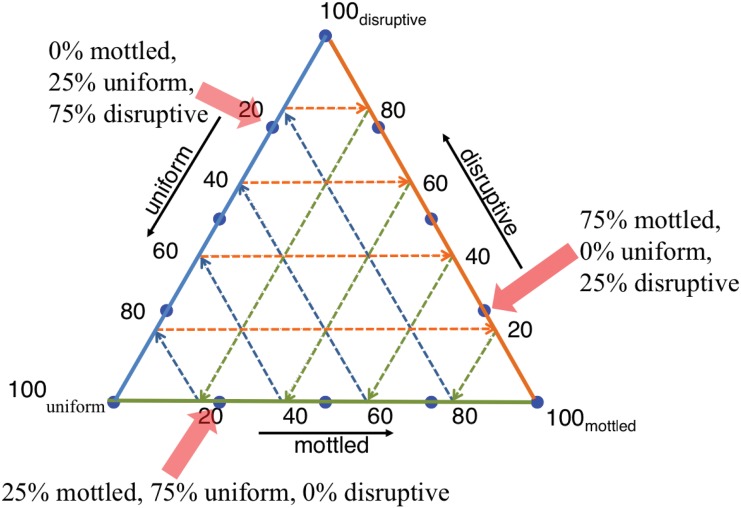
Example ternary plot showing the locations of camouflage categories (blue dots along the perimeter). The triangle vertices indicate categories that include only one camouflage pattern. Examples of mixed categories are indicated with the translucent red arrows. Categorizations that are mixtures of mottled and disruptive are along the right triangle perimeter. Classifications that are mixtures of uniform and disruptive coloration are along the left perimeter. Categorizations that are mottled and uniform are long the bottom perimeter. See the text for further information on interpreting ternary plots.

We investigated cuttlefish responses to bottom and side stimuli using complementary methods. We used ternary plots to categorize the cuttlefish responses (Fig 4). This type of graph allows the representation of data into three categories that sum to a constant value, in this case 100%. The vertices of the triangle represent camouflage categorizations of 100% mottled (bottom right vertex), 100% disruptive (top vertex), and 100% uniform (bottom left vertex). The percentage in a particular category decreases linearly with increasing distance from the corresponding 100% vertex. The percentages in the other categories are determined by following the point of interest along or parallel to the dashed internal grid lines (Fig 4). For example, to determine the percent disruptive, move from the point of interest toward the right side of the triangle, along or beside the grid lines parallel to the bottom. To determine the percent mottled, move from the point of interest toward the bottom of the triangle, parallel to the grid lines that form a 60° angle with the triangle bottom. The percent uniform is determined by moving toward the left side of the triangle, following the grid lines that form a 120° degree angle with the bottom.

Because of our chosen classification scheme, all of the original camouflage categorizations are on the perimeter of the ternary plot. The right side of the ternary plot includes categorizations of images with some percentage of mottled and disruptive camouflage patterning. The left side of the ternary plot includes categorizations of uniform and disruptive camouflage, and the bottom of the ternary plot includes images that are uniform and mottled. We also performed Kruskal-Wallis tests separately for the percent mottled, disruptive, and uniform in the cuttlefish responses to examine if there were differences in camouflage display when changing the bottom stimuli and when changing the side stimuli.

## Results

### Comparing responses within bottom stimuli

**Fig 5:**
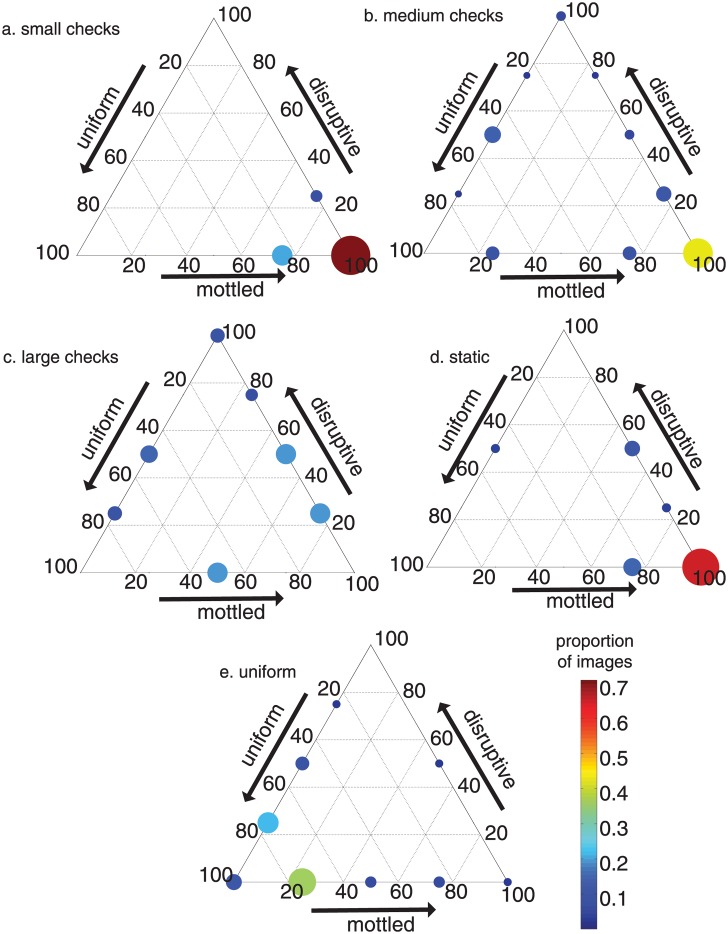
The classification of responses of cuttlefish exposed to different bottom stimuli. See text and Fig 4 for an explanation on how to read ternary plots. The larger the dot and warmer the color, the greater the proportion of images in that category. Proportion of cuttlefish images exposed to a bottom stimulus of (a) small checkerboard, (b) medium checkerboard, (c) large checkerboard, (d) television static, and (e), uniform.

Cuttlefish exposed to a small checkerboard bottom stimulus exhibited only 3 of the 12 possible camouflage categories (in order of decreasing frequency): 100% mottled, 75% mottled and 25% uniform, and 75% mottled and 25% disruptive (Fig 5a). The 100% mottled category was the most common, containing nearly 3/4 of the cuttlefish responses to a small checkerboard bottom stimulus.

Among cuttlefish exposed to a medium checkerboard bottom, the camouflage responses were more varied (Fig 5b). Similar to the small checkerboard bottom results, 100% mottled contained the greatest proportion, around 2/5, of the cuttlefish camouflage responses. However, the cuttlefish responses to the medium checkerboard bottom were also categorized into every possible combination of mottled and disruptive coloration and also every combination of uniform and disruptive body patterning (Fig 5b). The animals less frequently showed mixed patterns of uniform and mottled coloration.

Among animals responding to a large checkerboard bottom stimulus, again the classification was varied (Fig 5c). The most common camouflage categories were 75% mottled and 25% disruptive, 50% each of mottled and disruptive, and 50% mottled with 50% uniform. Each of those camouflage categories contained nearly 1/5 of the cuttlefish responses to a large checkerboard bottom.

The cuttlefish responses to a television static bottom (Fig 5d) were similar to the responses to the small checkerboard bottom stimulus (Fig 5a); most categorizations contained a relatively large percentage of mottled. In fact, the cuttlefish responses to the television static bottom were most often (~2/3 of the time) classified as 100% mottled (Fig 5d). However, unlike the cuttlefish response to the small checkerboard, a small proportion (~1/25) of cuttlefish responses were 50% uniform, 50% disruptive patterns.

The cuttlefish responses to a uniform bottom were unique in that they most frequently (~ 9/10 of responses) had a relatively large (50–100) percentage of uniform coloration patterning (Fig 5e). The camouflage category that contains the highest proportion (~1/3) of cuttlefish responses was 75% uniform and 25% mottled.

### Comparing responses within side stimuli

**Fig 6:**
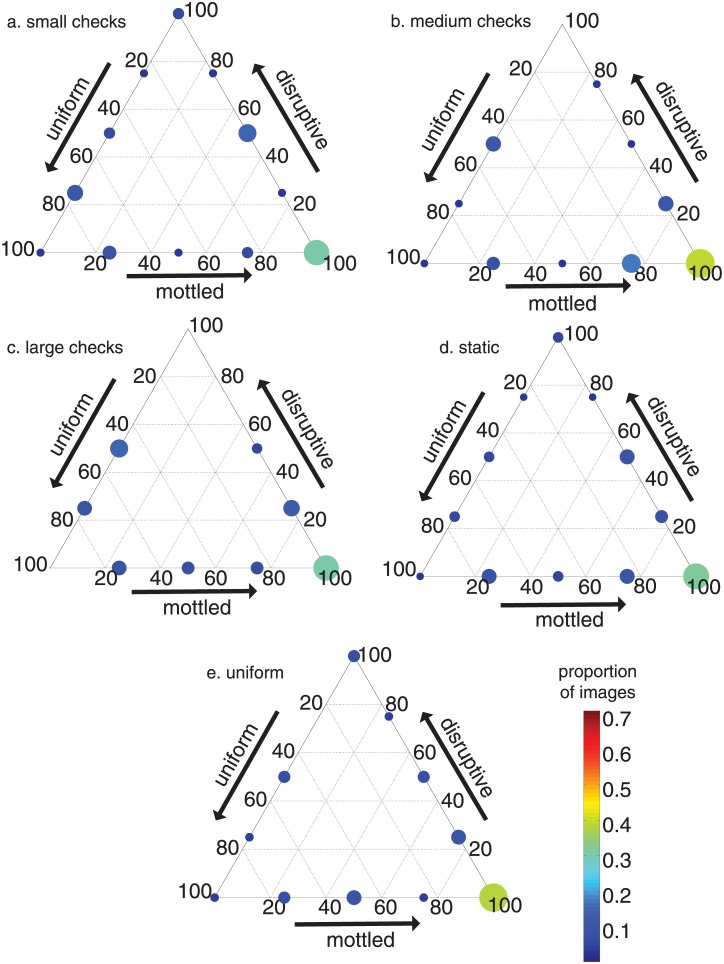
The classification of responses of cuttlefish exposed to different side stimuli. See text and Fig 4 for an explanation on how to read ternary plots. The larger the dot and warmer the color, the greater proportion of responses in that camouflage category. Proportion of cuttlefish responses to a vertical stimulus of (a) small checkerboard, (b) medium checkerboard, (c) large checkerboard, (d) television static pattern, and (e) uniform color.

The responses of cuttlefish to different side stimuli were not strongly distinct from one another (Fig 6). The 100% mottled category contained the highest proportion (from ~1/3 to 2/5) of cuttlefish responses to each of the 5 vertical stimuli. Furthermore, the cuttlefish responses within a stimulus group were relatively varied; nearly every possible camouflage category was expressed for each stimulus projected on the tank sides (Fig 6). For example, the animals responded to the large checkerboard by displaying 8 out of the possible 12 camouflage categories (Fig 6c). The response of the cuttlefish to the small checkerboard and television static side stimuli were classified into all 12 camouflage categories (Fig 6a and 6d).

### Comparing between bottom and side stimuli

**Fig 7:**
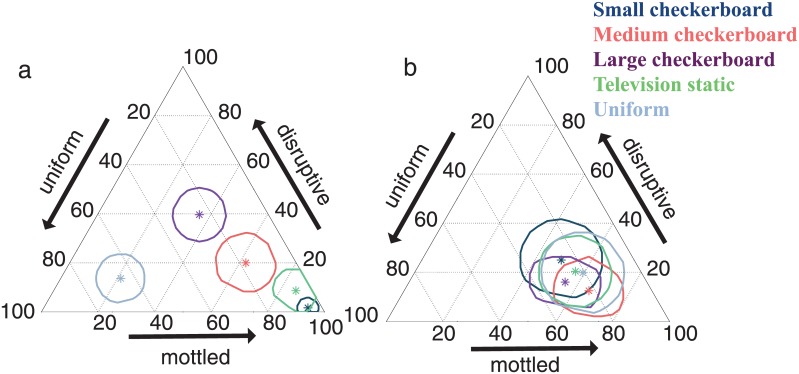
Average response of cuttlefish to each of the five stimulus patterns. The asterisk indicates the average value, and the surrounding shapes are the bootstrapped 95% confidence intervals. See text and Fig 4 for information on reading ternary plots. (a) Average response to different bottom stimuli. (b) Average response to different side stimuli.

To aid comparisons across the responses to the bottom and side stimuli, we calculated the center of mass of the cuttlefish responses to each bottom and side stimulus pattern (Fig 7). That is, for a given stimulus pattern, we calculated the average percent mottled, disruptive, and uniform out of all the camouflage responses (Table 1). Because no average value was zero for disruptive, mottled, nor uniform, all mean values were in the interior of the ternary plots. We also calculated the bootstrapped 95% confidence intervals (see Materials and Methods for details on this method) for these mean values, which reflect both the variability in responses and the number of images.

**Table 1:** Average percent classification of disruptive, mottled, and uniform for each of the bottom and side stimuli treatments.

Stimulus treatment	Average percent mottled (upper, lower 95% confidence interval)	Average percent disruptive (upper, lower 95% confidence interval)	Average percent uniform (upper, lower 95% confidence interval)
Bottom small checkerboard	93% (88, 97%)	2% (0, 6%)	5% (1, 10%,)
Bottom medium checkerboard	64% (51, 77%)	17% (6, 29%)	19% (8, 31%)
Bottom large checkerboard	33% (22, 44%)	42% (31, 53%)	25% (14, 36%)
Bottom television static	83% (73, 93%)	10% (1, 19%)	7% (0, 15%)
Bottom uniform	20% (8, 32%)	20% (9, 33%)	60% (47, 72%)
Side small checkerboard	48% (29, 67%)	30% (11, 49%)	22% (4, 41%)
Side medium checkerboard	63% (46, 79%)	14% (1, 30%)	23%, (9, 38%)
Side large checkerboard	54% (38, 71%)	18% (6, 30%,)	28% (14, 43%)
Side television static	59% (43, 76%)	14% (1, 28%)	27% (11, 43%)
Side uniform	59% (44, 73%)	23% (9, 38%)	18% (5, 32%)

When we compared the average camouflage responses to the bottom stimuli, some general patterns arose. The average response to the small checkerboard bottom was 93% mottled (88%, 97% upper and lower 95% confidence intervals) (Fig 7a, Table 1) while disruptive and uniform patterning made up 2% (0%, 6%) and 5% (1%, 10%) of the average classification, respectively (Table 1). For the medium checkerboard, the response was 64% (51%, 77%) mottled but also included nearly equal percentages of disruptive and uniform patterning (Fig 7a, Table 1). For the large checkerboard, the average was 33% (22, 44%) mottled, 42% (31, 53%) disruptive, and 25% (14, 36%) uniform. Given the lack of overlap in the confidence intervals shown in Fig 7a among the small, medium, and large checkerboard averages, the camouflage classifications of animals exposed to these different bottom stimuli were significantly distinct.

The average response to the television static bottom stimulus was 83% (73, 93%) mottled, 10% (1, 19%) disruptive, and 7% (0, 15%) uniform. This average was not significantly different from the small checkerboard average (Fig 7a). The mean response to the uniform bottom was approximately 60% (47, 72%) uniform with nearly equal percentages of mottled and disruptive patterning.

**Fig 8:**
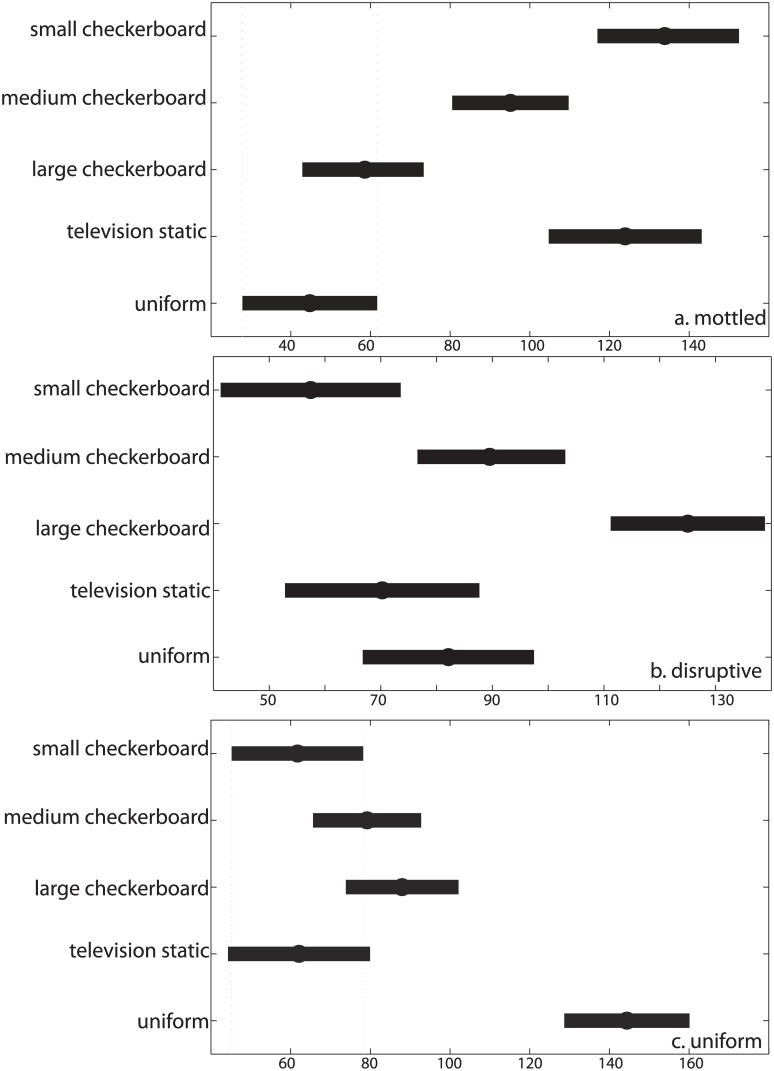
Comparison of individual components of camouflage response to bottom stimulus patterns. (a) percent mottled, (b) percent disruptive, and (c) percent uniform. The dots represent means and bars represent standard deviations. An overlap of the lines in each panel indicates that the groups were not significantly different based on a post-hoc test using the Dunn-Sidak approach.

Based on the results of the Kruskal-Wallis test, there were statistically significant differences in the percent mottled coloration among different bottom stimuli, χ^2^(4) = 81.30, p <<0.001. Post-hoc comparisons of the percent mottled response using a Dunn-Sidak approach (Fig 8a) showed that the small checkerboard, medium checkerboard, and television static stimulus groups (M = 133.9, 95.1, 123.9, respectively, SD = 9.0, 7.3, 9.6, respectively) were significantly different than the large checkerboard and uniform stimulus groups (M = 58.6, 44.9, respectively, and SD = 7.7, 8.5, respectively). Small checkerboard and medium checkerboard groups were also different. All other comparisons were not statistically different.

Comparing separately the percent disruptive and percent uniform camouflage response, there were also significant differences among groups of different bottom stimuli, χ^2^(4) = 47.10 and 70.47, respectively, and p <<0.001 for both. Similar post-hoc tests to those described above indicated that the disruptive response to a large checkerboard bottom (M = 125.1, SD = 7.0) was statistically significant from all other groups (all means ≤ 89.2, SD ≤ 8.7), and the response to the medium checkerboard (M = 89.2, SD = 6.6) was distinct from the small checkerboard group (M = 57.4, SD = 8.1; Fig 8b). All other comparisons were not significantly different. The post-hoc test for the percent uniform response showed that the group exposed to the uniform bottom (M = 144.4, SD = 7.9) was statistically significantly different from all other groups (all means ≤ 88.0, SD ≤8.9), and all other comparisons were not different (Fig 8c).

When we compared the average responses to each of the side stimuli, the mean values did not differ significantly from one another (Fig 7b). All means were between ~50% and 65% mottled, ~15% and 30% disruptive, and between ~15% and 30% uniform (Table 1). Therefore, mottled made up the greatest percentage of the average response to each side stimulus pattern.

A Kruskal-Wallis test comparing percent mottled responses among groups of side stimuli failed to reject the null hypothesis that there were no differences among responses to the side stimulus patterns, χ^2^(4) = 2.81, p = 0.59. Separate comparisons of the percent disruptive and percent uniform responses to different side stimuli also failed to reject the null hypothesis, χ^2^(4) = 3.09 and 1.70, respectively, p = 0.54 and 0.79, respectively.

## Discussion

The cuttlefish *S*. *officinalis* responded differently to each of the five visual stimuli used in this study. The average responses to the *bottom* stimuli are more distinct from one another than the camouflage patterns expressed for the different *side* stimuli (Figs 7 and 8). These results indicate that, in our experimental configuration in which the animals are not allowed directly adjacent to the tank walls, the cuttlefish camouflage responses are influenced more by the bottom than the side stimuli. Because it is the bottom that elicits distinct results, we focus foremost on the cuttlefish responses to those visual stimuli.

Previous work suggests that cuttlefish respond to high contrast patterns either with mottled coloration for small, high-density patterns such as squares in a checkerboard pattern less than 40% of the animal’s White square component (a light, rectangular feature on the dorsal area of the mantle (Hanlon and Messenger, 1988)), or with disruptive patterning for checkerboard sizes between 40 and 120% of the animal’s White square [6]. In this study, we found corroborating results: as the checkerboard pattern size increases, the cuttlefish responses include increasingly large colored patterns and components (Figs 5a–5c, 7a and 8b).

The cuttlefish response to the television static stimulus is similar to that for the small checkerboard pattern when simultaneously considering all body patterning responses, (Figs 5a and 5d and 7a) and also when comparing the percent mottled, disruptive, and uniform separately (Fig 8). That is, cuttlefish largely responded with a mottled pattern when exposed to the television static pattern on the bottom of the tank. This result is not entirely anticipated given previous work. The television static stimulus pattern has lower contrast and different spatial resolution of contrasting units compared to the checkerboard backgrounds and thus may be expected to elicit uniform coloration [9,10]. However, the contrast in the television static pattern was apparently high enough and spatial frequency low enough to elicit mottled coloration.

When the animals were exposed to a uniform grey bottom, they preferentially showed a uniform pattern, albeit with some variability (Figs 5e and 7a). When focusing solely on the percent uniform in cuttlefish responses to the different stimulus patterns, the uniform stimulus group is distinct from all others (Fig 8c). These findings are in agreement with previous studies reporting uniform coloration for cuttlefish shown low-contrast and uniformly colored stimuli [3,4,18].

For each of the five side stimulus patterns, the camouflage display was often highly mottled (Figs 6 and 7b). The lack of distinct patterns when varying the side stimulus compared to the distinct patterns when changing the bottom stimulus (Fig 7) supports the idea that animals are responding predominantly to the bottom of the tank. The greater sensitivity to the bottom stimulus can also help explain the average responses to the side stimuli (Fig 7b). Three of the five stimulus patterns—the small and medium checkerboard pattern and television static—typically elicit highly mottled coloration when placed on the bottom of the tank (Fig 7a). The static stimulus group is also not statistically significantly different from the small and medium check stimulus groups when comparing separately the percent mottled, disruptive, and uniform in cuttlefish responses (Fig 8). Therefore, if the animals respond more to the bottom than the sides of the tank, regardless of the stimulus on the side of the tank, the majority of camouflage responses should be highly mottled, which is what is shown in Fig 7b.

Several previous studies, examined the response of cuttlefish to side stimuli [1,18–20]. In all studies, with the exception of Hanlon and Messenger [1], cuttlefish were found to respond to these side stimuli. Barbosa et al. [18] found cuttlefish to respond to both bottom and side stimuli. Most importantly in the context of this study, checkerboard patterns displayed on the side were sufficient to elicit a disruptive response. Furthermore, Ulmer et al. [20] found that checkerboard patterns on 3-dimensional objects or walls were more influential than bottom stimuli in eliciting a disruptive pattern. In this study, however, cuttlefish of the same species responded more strongly to the bottom substrate.

We attribute the variation in responses to the proximity of the animal to the side stimulus. For instance, Barbosa et al. [18] note that in the majority of their experimental treatments, the animals showed a unilateral or asymmetrical response of at least one coloration component that constitutes disruptive camouflage. They attribute such results to the “wall effect” because the animals were most often found against the wall of the tank. Also, the strongest response to vertical plane stimuli described by Ulmer et al. [20] occurred when the checkerboard pattern was close to the tank bottom rather than placed higher up. Although Ulmer et al. [20] also found cuttlefish to preferentially respond to the sides rather than the bottom of the arena when they were completely covered in checkerboard patterning, they note that their own previous experiments showed bottom checkerboard patterns to elicit disruptive patterns, as have many others (e.g., [4,6,9,22]). In the study by Buresch et al. [19] in which animals were found to masquerade as high contrast 3D objects, the object was always placed in the center of the arena, within reach of the cuttlefish.

In contrast to these studies, the animals in these experiments were restricted from settling near the tank walls. Thus, we suggest that when cuttlefish are allowed next to tank walls or 3D objects, they background match to or masquerade as these lateral visual stimuli. However, when these animals are kept away from the stimulus walls or objects, they preferentially respond to the bottom stimulus. While currently not possible in the Sub Sea Holodeck, future experiments could further examine whether or not proximity to the visual stimulus helps drive cuttlefish response by allowing the animal to be next to the tank wall and on top of a glass bottom with a pattern further below or by varying the combinations of distances from the bottom and sides of the tank. The cuttlefish could then be examined to see if they respond to the sides instead of the distanced bottom stimulus pattern.

It is currently possible in the Sub Sea Holodeck to examine the influence of the magnification of the side stimulus patterns on the cuttlefish responses. Proximity to the stimulus patterns influences the size of the patterns and thus initially could be considered an important factor. However, in light of the lack of response of cuttlefish to the side stimulus patterns already presented, these results were not statistically significant and thus are presented in the supporting information (S1 Supporting Information).

We also acknowledge that the responses of cuttlefish to the bottom rather than the sides of the tank may be due to the different media used (see also the *Experimental Setup* in the Materials and Methods section below). That is, the bottom stimuli were produced using laminated paper printouts, while the side stimuli were displayed on plasma screens. These different media may have led to the preferential response of the cuttlefish to the bottom rather than side stimuli. However, there is no reason to believe that the stimulus patterns themselves, and thus the cuttlefish camouflage patterns, vary with the media used to display the stimuli. For example, the cuttlefish visual acuity is approximately 8 cycles per degree [23]. The pixel width in our screens is ~0.0485 cm. Because the animals were in the center of the tank, the viewing distance was ~50 cm. Thus, one pixel is about 0.06 degrees, which is about 18 cycles per degree. Therefore, the animals would require more than twice the visual resolution they have to distinguish one pixel. Even if the animal were on the edge of the acrylic tube rather than the exact center of the tank, the viewing distance would be 28 cm. This viewing distance would result in ~10 cycles per degree, which is again higher than what the animal can see. While there may be a slight magnification of the plasma screens due to the tank, water, and acrylic tube, we still argue that the images in the screens are indistinguishable from those in the printed poster. Furthermore, the radiance of the sides and bottom are roughly equal, and while the spectral quality may be slightly different, the effect is likely negligible, especially because cuttlefish are colorblind.

Another possible concern is the flicker frequency of the plasma screens. The plasma screens used in the Sub Sea Holodeck refresh with a frequency of 600 Hz. Cuttlefish have a fusion frequency of 30 Hz [24] and thus are not aware of the plasma screen flicker.

Given these reasons, we believe that the most reliable behavior with the least amount of human disturbance resulted from our use of the chosen media on the bottom and the sides of the tank. Future studies could include a more thorough investigation of cuttlefish responses to different media, as well as the orientation of the stimulus patterns and a greater variety of distances of the specimen to the stimuli.

While this and other related experiments were done in a laboratory setting, it is important to think of these responses in the context of the natural environment. Cuttlefish, particularly young ones, are largely benthic. In an open area, a cuttlefish’s best defense against predators may be to camouflage with the bottom substrate. Once vertical structures become closer, it may be more advantageous to masquerade or resemble vertical structures to avoid predator detection from both a top-down and side view [20].

Future studies could examine, both in the laboratory and in the wild, the distance between cuttlefish and side stimuli to see if there is some threshold distance inside of which cuttlefish preferentially respond to the vertical plane stimuli, and how plastic this response may be with different ages and species of cuttlefish. With such knowledge, we will have a better understanding of what visual cues drive the dynamic adaptive coloration of cuttlefish in their diverse environments.

## Materials and Methods

### Sub Sea Holodeck

Our study was conducted using a novel aquarium environment called the Sub Sea Holodeck (Fig 2 [21]), referred to hereafter as the Holodeck. The Holodeck is an aquarium tank that is mounted inside a fiberglass frame with plasma or DLP displays behind all surfaces (side and bottom, respectively) that can display visual content. The tank itself is made of acrylic and measures 101 x 101 x 66 cm to accommodate commercially available screens. The Holodeck also contains 4 high-speed, waterproof USB cameras that allow the activities of the residents inside the tank to be recorded without disruption. While the video screens have the capability of being controlled with custom software (see [21] for additional information]), in this study they were manipulated via Matlab version R2013a.

### Cuttlefish husbandry

The cuttlefish *Sepia officinalis* used in this study are not an endangered or protected species. Fisherman collected their eggs per request in the wild off the lines of fish pots. There was no need for permission for collection because it occurred in open fishing grounds, and the eggs were otherwise going to be discarded upon fish pot retrieval. The retrieved eggs were hatched at a facility in New York. Approximately one month after hatching, the animals were shipped to and reared at Duke University. The ethics committee that approved the work was the Institutional Animal Care and Use Committee at Duke University. The protocol number for this study is A173-11-07. This work was carried out in strict accordance of all essential ethical requirements and care was taken to minimize any animal discomfort. Specifically, the cuttlefish were housed in a ~ 950L recirculating, artificial seawater system maintained at 18°C and 32 ppt salinity. Temperature and salinity were monitored and adjusted daily and other water parameters including ammonia, nitrite, nitrate, and pH were monitored and adjusted twice per week to ensure water quality. Cuttlefish were fed one to two times daily using a mixed diet of live shrimp, frozen shrimp, frozen scallops, and frozen silversides.

Prior to our experiments, the animals were monitored daily for behavioral anomalies, signs of stress or injury, and normal feeding habits. Immediately before testing, the animals were observed to ensure activity level, respiration rate, and stress levels were within the acceptable range of normal observed behavior. In all cases, extra care was taken while transferring animals into the experimental tank and extra time was allowed for acclimation prior to beginning the experimental run. During experiments, the animals were continually observed via a live web cam feed for signs of stress and agitation. The only adverse effects observed were that, occasionally, an animal would ink during transfer into the experimental tank, during the acclimation process, or during the experimental runs. Following experiments, the cuttlefish were again observed for signs of stress, injury, and resumption of normal behavior.

### Experimental set up

The experiments were conducted when the animals were approximately 6.5 months old and about 10 cm mantle length. Seven individuals of the species *Sepia officinalis* were used for our study. The number of individuals utilized during the experiments was based on the availability of healthy, stress-free animals at the time the experiments were performed.

Cuttlefish were exposed to 5 different stimulus patterns: uniform grey, television static, as well as small, medium, and large checkerboard patterns (Fig 1). The squares in the small checkerboard patterns were 2 cm, the medium checkerboard 4 cm, and the large checkerboard 7 cm, which had an angular size of 2.3, 4.6, and 8 degrees, respectively, when viewed from the correct distance (the center of the aquarium). The television static pattern was included to expand the repertoire of stimulus patterns beyond those commonly used in previous work (e.g., [14,15,20]). In total, there were 25 different combinations (5 bottom x 5 side) of bottom and side patterns. Of the 7 cuttlefish used, 4 were exposed to all 25 different combinations of bottom and side patterns. The other 3 cuttlefish were only exposed to between 5 and 20 of the stimulus combinations because they died before we could expose them to all the combinations.

Laminated paper printouts of the background patterns were used on the bottom of the tank. 42-inch high-definition 1920 by 1080 Panasonic plasma screens were used to present the side stimuli. While different media may differentially affect cuttlefish responses, preliminary experiments indicated that the light emitting from the bottom, but not the side, screens disturbed the animals, which were subsequently incapable of eliciting a systematic response. Therefore, while not ideal, we believe more reliable behavior resulted from the use of laminated paper printouts on the bottom of the tank, while the plasma side screens lessened human interference with the animals as much as possible. (See the Discussion section for further information on the different display media.)

After filling the aquarium with artificial seawater, a bottom pattern, chosen with a random number generator, was put in place. The tank was connected to a closed-loop, recirculating artificial seawater system. Once the bottom pattern was in place, a circular acrylic tube (45 cm in diameter) was placed in the center of the tank. A circular enclosure was chosen to lessen shadows and prevent settling in corners. Subsequently, one cuttlefish specimen was placed in the middle of the arena, and the stimulus, one of the five patterns again chosen with a random number generator, gradually appeared simultaneously on each side screen, going from zero to maximum intensity in 5 seconds, following an s-shaped curve. For each new visual environment, the animal was allowed approximately 10 minutes to acclimate, which was determined by a cessation of swimming and staying in one location for at least two minutes. After the settling period, the animal was photographed with a Nikon D700 camera with a Nikon AF-S 24–120 mm f/4G ED VR lens. Other cameras were used for ancillary information, which was not used in this study. Two diffuse halogen spotlights reduced shading artifacts in the images and allowed the bottom pattern’s luminescence to be similar to that of the plasma screens. The camera settings were retained and identical throughout all runs.

Each animal was photographed a minimum of 17 times but typically 25 times for a particular bottom and side stimulus combination. Each photograph was taken ~10 seconds apart. After photographing, the side displays faded back to black, a new stimulus was randomly chosen, and the process was repeated.

### Image analysis

To mitigate the likelihood of autocorrelation, we used the two usable (i.e., non-blurry) images that were furthest apart in time among the replicate images for each individual in a given treatment. Each cuttlefish image was segmented into a standardized oval template with no background stimulus visible. Only images that had the background completely removed were used. That is, during the image analysis, the classifier had no knowledge of what the bottom nor side stimuli were. Some sets of images were blurry or could not be fit properly to the template without significant distortion; such images were not used in our analyses. In all, 175 images were used. Each image was randomly displayed and manually categorized as a percentage of mottled, disruptive, and/or uniform. To simplify the classification process, cuttlefish were classified into at most 2 of the 3 general camouflage types of mottled, disruptive, and uniform. Camouflage classification was done in 25% increments (i.e., 0, 25, 50, 75, or 100%). That is, images could be classified as 100% mottled, 75% mottled and 25% disruptive, 50% mottled and 50% disruptive, etc. In all there are 12 categories, which we refer to as camouflage categories. The classification into each camouflage category was based both on the intensity of body patterning displayed and on the surface area of cuttlefish covered with a general camouflage type.

Because of the importance of consistency in classification, the original observer re-classified a subset of 69 images over a year after the original scoring. Cohen’s κ was calculated to determine the level of agreement in classification at these different time periods. κ was 0.6350 (95% CI, 0.50, 0.77), p <<0.001, which, according to the benchmarks provided in Landis and Koch [25], indicates a substantial agreement in classification.

In this classification scheme, we have compromised between completeness and simplicity. We have attempted to include enough categories to encapsulate the diversity of cuttlefish body patterning but also avoided too many categories, which may make the classification process cumbersome, complicated, and potentially inaccurate. Each image was categorized using a classification guide with examples of the possible camouflage category (Fig 3). This method, while subjective, has similarities to the manual grading methods of Chiao et al. [9] and Mäthger et al. [14] and the method of creating a lab manual mentioned by Mäthger et al. [3]. This classification technique also has the advantage of allowing an investigation of the mottled, disruptive, and uniform responses, rather than just the extent of disruptiveness as has often previously been done (e.g., [3,7,18]). Furthermore, any biases resulting from the classification scheme used in this study are consistent across all classifications, despite the bottom or side stimulus, as indicated above. Therefore, a relative comparison of the cuttlefish responses to either the bottom and side stimuli can still be made.

### Comparing the effect of bottom versus side stimuli

After each cuttlefish response was classified into one of the twelve camouflage categories, all the responses were divided into five groups based on the five bottom stimulus patterns (Fig 1). Within each group, we calculated the proportion of cuttlefish responses in each of the twelve categories. For example, out of all the cuttlefish responses to the television static bottom, we calculated the proportion of those responses in the 50% mottled, 50% disruptive camouflage category. We followed this same methodology for the side stimulus patterns. That is, using all of the cuttlefish responses, we calculated the proportion of responses in each of the twelve camouflage categories for each of the five side stimulus groups. Comparisons of the proportion of responses in each of the camouflage categories were then made within and across each of the groups.

To more easily compare responses to stimulus patterns, we calculated the center of mass for the responses to each of the bottom and side stimuli (see Fig 7 and also the Results section). That is, using the proportion of responses in each of the twelve categories for each stimulus, we calculated the average response. To quantify the error, we calculated 95% bootstrap confidence intervals. That is, out of all the responses to a given bottom or side stimulus, we resampled with replacement from those responses to obtain a subsample of the same size as the original sample. We repeated this process 10,000 times to obtain 10,000 subsamples of the responses to each of the bottom stimuli and each of the side stimuli. We then calculated the average response for each of those subsamples, and the 95% of average responses that were closest to the original sample mean were used to delineate the bootstrapped confidence intervals shown in Fig 7.

Due to the nature of the data (compositional with structural zeros), we used a separate Kruskal-Wallis test for the percent mottled, disruptive, and uniform in camouflage responses to test if there were significant differences among bottom stimuli groups and side stimuli groups. When appropriate, we used a post-hoc test with a Dunn-Sidak correction to investigate which groups were significantly different.

## Supporting Information

S1 Supporting Information Cuttlefish responses to side stimulus magnifications. (DOCX)
