# Supplementary material for: Cuttlefish Sepia officinalis Preferentially Respond to Bottom Rather than Side Stimuli When Not Allowed Adjacent to Tank Walls
Source: PLoS One. 2015 Oct 14;10(10):e0138690. doi: 10.1371/journal.pone.0138690 (PMC4605735; doi:10.1371/journal.pone.0138690)
Supplement: S1 Supporting Information — (DOCX) [file pone.0138690.s001.docx]

**S1 Supporting Information. Cuttlefish responses to side stimulus magnifications.**

We examined the response of the cuttlefish to various magnifications of the side stimuli. That is, we investigated the percent disruptive response when displaying simultaneously on all four sides of the tank the same stimulus pattern that was magnified to be 100, 200, 400, and 600% its original size. Because of unequal sample sizes and the ordinal and compositional nature of the dependent variables, we used a Kruskal-Wallis test to examine statistical differences in disruptive response among the different levels of magnification for each appropriate (i.e., non-uniform) stimulus pattern separately (S1 Table).

There was no significant difference at the α = 0.05 level in percent disruptive response with change in magnification for any of the side stimulus patterns. For the sake of completeness, we performed the same set of tests to look individually for changes in the percent mottled and percent uniform of the cuttlefish responses. For each stimulus pattern examined separately, there was no significant difference in either percent mottled or percent uniform camouflage response with varying magnification (S1 Table). The lack of differences in camouflage response to different magnifications of the side stimuli further suggests that the animals are not responding to the projections on the tank sides.

S1 Table. Results from Kruskal-Wallis test comparing the responses of cuttlefish to different magnifications of the side stimulus patterns.

| Stimulus | Sm check | Md check | Lg check | TV Static |
| --- | --- | --- | --- | --- |
| p value for mottled | 0.3289 | 0.8425 | 0.8023 | 0.4415 |
| χ^2^ statistic for mottled | 3.44 | 0.83 | 1.00 | 2.69 |
| p value for disruptive | 0.3091 | 0.6025 | 0.3677 | 0.3885 |
| χ^2^ statistic for disruptive | 3.59 | 1.86 | 3.16 | 3.02 |
| p value for uniform | 0.82 | 0.93 | 0.96 | 0.86 |
| χ^2^ statistic for uniform | 0.90 | 0.44 | 0.31 | 0.76 |
